# Isolating viable bacteria from positive blood cultures: impedance cytometry analysis

**DOI:** 10.1007/s10544-026-00847-5

**Published:** 2026-10-01

**Authors:** Daniel Nathan Tomás Cançado, Max Seidy Saito, Daniel Spencer, Hywel Morgan

**Affiliations:** 1 School of Electronics and Computer Science, University of Southampton, Southampton, SO17 1BJ UK; 2 School of Cancer Sciences, University of Southampton, Southampton, SO17 1BJ UK; 3 Institute for Life Sciences, University of Southampton, Southampton, SO17 1BJ UK

**Keywords:** Antimicrobial resistance, Bloodstream infections, Sepsis diagnostics, Rapid phenotypic antimicrobial susceptibility testing, Microfluidic impedance cytometry, Optical flow cytometry, Single-cell analysis, Bacterial viability, Microfluidics, Electroanalytical methods

## Abstract

Bloodstream infections (BSIs) lead to sepsis, an acute inflammatory response associated with high mortality. Current clinical treatment pathways rely on culture-based diagnostics to identify the infectious agent and determine its susceptibility to antibiotics, delaying targeted therapy. Recent approaches to accelerate this focus on the isolation of bacteria directly from blood cultures for rapid downstream analysis using haemolytic agents to replace the gold-standard purity plate subculture. However, many of these methods impact bacterial viability during the early stages of growth. Rapid phenotypic antimicrobial susceptibility testing requires a pure inoculum of actively dividing bacteria to deliver early results (within 3 h), but most isolation chemistries are developed for mass spectrometry identification which does not require viable bacteria. This is particularly relevant for Gram-positive pathogens, as these are more susceptible to detergent agents used for haemolysis. This study investigated five different blood lysis protocols for the isolation of two different bacteria; *Escherichia coli* (Gram-negative) and *Staphylococcus aureus* (Gram-positive) from positive blood bottles. Bacterial viability was determined with microfluidic impedance cytometry and standard colony counting, which was also used to determine the extraction efficiency. For all five protocols, the numbers of extracted bacteria were generally high for the two organisms when determined with colony counting (following overnight incubation). However, when determined immediately after extraction with impedance cytometry, the counts were lower for *S. aureus*. The bacterial viability was also investigated with optical flow cytometry immediately after extraction showing inhibition of *S. aureus* growth with Sepsityper^®^ but not with the milder detergent saponin.

**Graphical Abstract:**

**Figure:**
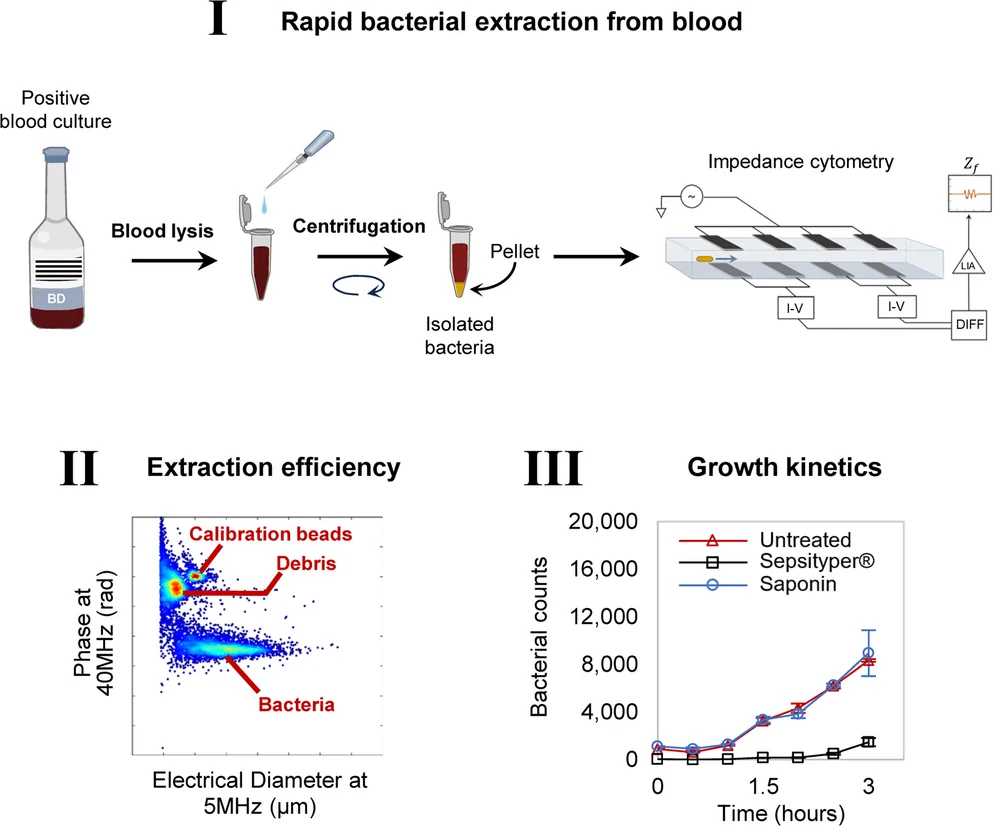

**Supplementary Information:**

The online version contains supplementary material available at https://doi.org/10.1007/s10544-026-00847-5.

## Introduction

Bloodstream Infections (BSIs) are a life-threatening condition that can rapidly progress into sepsis, an acute immune response to infection that leads to organ failure. The condition is associated with high mortality rates and is responsible for an increasing economic burden (Mancini et al. 2010). To reduce hospitalisations, patients with suspected BSIs are treated empirically with broad-spectrum antibiotics before information about the causative pathogen is acquired. Empiric antimicrobial therapy is often lifesaving because treatment is immediate, however its overuse has contributed to Antimicrobial Resistance (AMR), and AMR reduces the effectiveness of the available antimicrobial stock (Uddin et al. 2021). There is a need for targeted antimicrobial therapy, combined with rapid diagnostics to identify the pathogen and to determine the optimal antimicrobial early (GBD 2021 Antimicrobial Resistance Collaborators 2024).

**Fig. 1:**
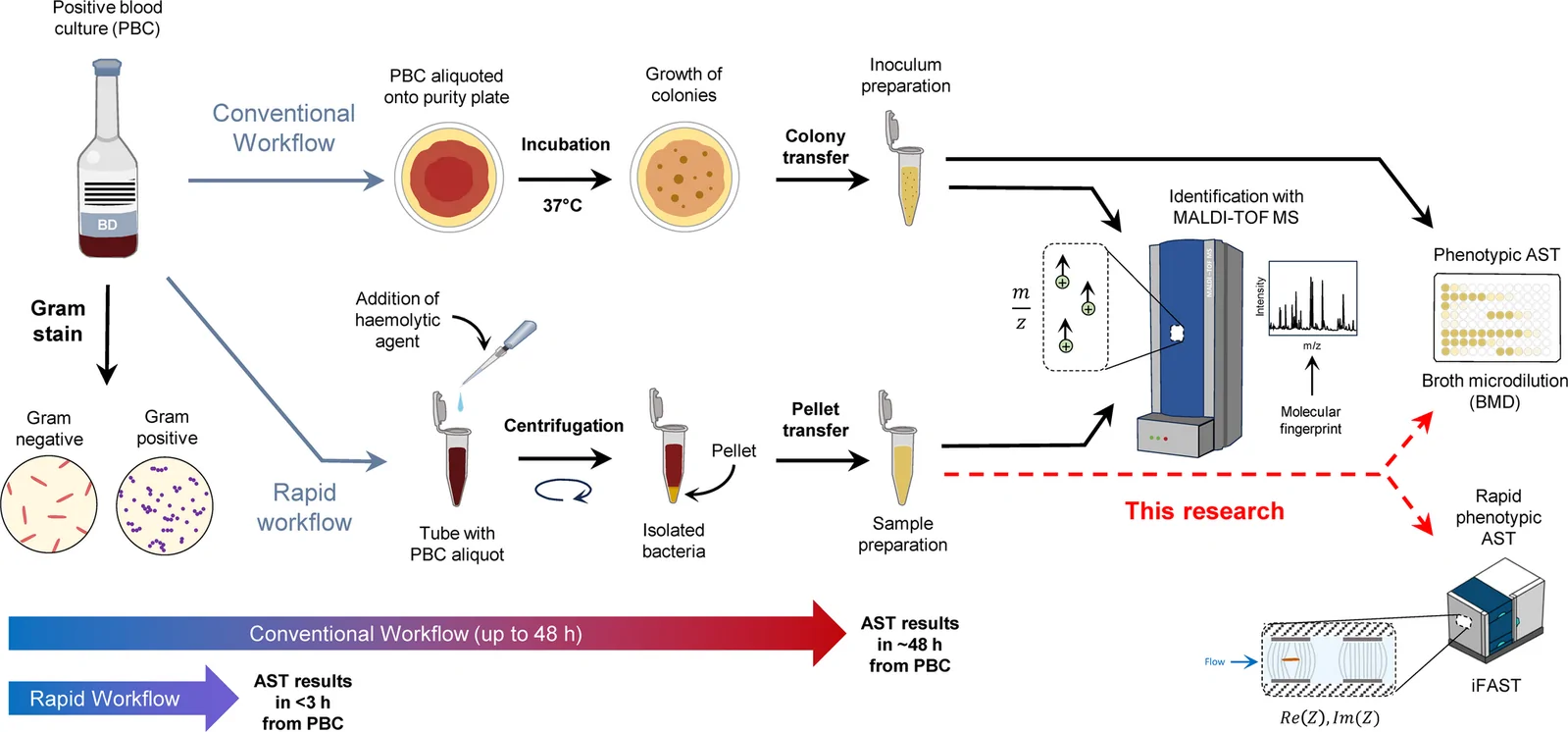
Example clinical workflow for BSI diagnosis. A PBC is processed for pathogen identification by MALDI-TOF MS followed by a phenotypic AST. The standard workflow requires a purity plate subculture prior to growth-based AST (~ 48 h). A rapid workflow replaces the subculture by isolating bacteria directly from the PBC using different extraction protocols, as done for MALDI-TOF MS. The dashed pathway indicates that the focus of this study is on direct isolation specifically for single-cell rapid phenotypic AST platforms

A typical diagnostic workflow is shown in Fig. 1. Patient blood is incubated in a blood bottle incubator to determine the presence of the pathogen (Opota et al. 2015). If a blood bottle flags positive, the bacteria are Gram-stained and a sample from the Positive Blood Culture (PBC) is then aliquoted onto solid growth media, then further incubated until isolated colonies are visible by eye (the purity plate subculture). This step is necessary because conventional identification and Antimicrobial Susceptibility Testing (AST) assays have been optimised to work with a monomicrobial suspension which has been prepared from a purity plate, rather than the direct use of the bacteria inside blood cultures; although there have been some recent advances with this approach (e.g. RAST (Jonasson et al. 2023)). Bacteria from purity plate colonies are identified using Matrix-Assisted Laser Desorption/Ionization Time-Of-Flight Mass Spectrometry (MALDI-TOF MS) and an overnight phenotypic AST is also carried out (e.g. broth-microdilution or disc diffusion). The entire workflow can take over 48 h to give actionable results (Banerjee and Humphries 2021, Wheat 2001, O’Neill 2016), and this turnaround time can be fatal because mortality for BSIs increases by 8% for every hour of delayed intervention (Kumar et al. 2006).

Rapid sample preparation protocols (10–30 min) are used to isolate bacteria from PBCs for identification using mass spectrometry (MALDI-TOF MS). The Sepsityper^®^ kit and related protocols (Weller and Maier 2011, Morgenthaler and Kostrzewa 2015, Ponderand et al. 2020, Schubert et al. 2011) isolate bacteria directly from PBCs for a MALDI ID. This is a proprietary lysis buffer containing ionic detergent(s) (Weller and Maier 2011, Tanner et al. 2017) that destroys the Red Blood Cells (RBCs), along with centrifugation (~ 16,000 × g) to pellet the bacteria and remove cellular debris. MALDI-TOF identifies samples based on protein/lipid analysis, and viable organisms are not required. However, for rapid phenotypic ASTs the organisms must remain viable and culturable after isolation. Identification scores for Gram-positive organisms have also been reported to be lower using Sepsityper^®^ (Morgenthaler and Kostrzewa 2015, Choi et al. 2025, Lin et al. 2022), suggesting that this lysis buffer may be suboptimal for these organisms. This is clinically relevant because Gram-positive pathogens account for over half of BSIs (Pfaller et al. 1999, Mendes et al. 2018), with *Staphylococcus aureus* being one of the most significant causes of infection.

There is therefore a need to develop milder sample extraction protocols for emerging rapid phenotypic AST workflows (Spencer et al. 2020, Silva-Dias et al. 2021, Kandavalli et al. 2022). For example, Pina-Vaz et al. (2024) used a non-ionic detergent lysis (Tergitol) with differential centrifugation to isolate both Gram-negative and Gram-positive organisms from blood demonstrating antimicrobial susceptibility results in under 2 h (FASTinov). Other mild detergents such as saponin or Triton X-100 have been used for both MALDI ID and phenotypic AST (Massey et al. 2022, Feng et al. 2013, Filbrun et al. 2022, Wang et al. 2015, Yonetani et al. 2016).

Several rapid phenotypic ASTs have been developed delivering results directly from PBCs within 6–8 h (Menchinelli et al. 2024, Funke and Funke-Kissling 2004, Anton-Vazquez et al. 2019, Charnot-Katsikas et al. 2018), including for example, Accelerate Pheno (Charnot-Katsikas et al. 2018), the Vitek Reveal (Menchinelli et al. 2024) and the Alfred 60 AST (Anton-Vazquez et al. 2019). However, these systems still require several hours to generate results as their workflows rely on continuous growth monitoring of bacteria. Novel techniques such as single-cell microscopy (Baltekin et al. 2017), bacterial motility imaging (Syal et al. 2016), microfluidic time-lapse imaging (Choi et al. 2013), Raman microscopy (Zhang et al. 2022), and electrical and electrochemical impedance (Spencer et al. 2023, Riester et al. 2024) are also being explored. The latter is particularly simple as it involves using electrical methods to measure the growth of bacteria with and without antibiotics, examples include the Microplate Dx RapidPlate (Hannah et al. 2019, Ralston et al. 2026).

We recently developed a label-free rapid AST using microfluidic impedance cytometry to analyse changes in the electrical properties of bacteria after a short exposure to antibiotics (Spencer et al. 2020). This test provides susceptibility data for bacteria extracted from a positive blood bottle within 2–3 h (Hornsey et al. 2026). However, the viability and culturability of bacteria after extraction from a PBC has not been widely investigated. This is important because rapid phenotypic ASTs require actively growing bacteria. In this paper we compare several different chemical lysis protocols adapted from the literature (Boardman et al. 2015, Boyd et al. 2015, Lee et al. 2018, van Assche et al. 2020), with the Sepsityper^®^ kit as a reference method. The effect on the viability of the isolated bacteria was determined for early stages of growth (1–3 h) and after 18 ± 2 h. For each protocol, a representative Gram-negative (*Escherichia coli*) and Gram-positive (*Staphylococcus aureus*) species was used, and the processed bacterial samples were analysed using both optical and impedance flow cytometry.

### Impedance-flow cytometry for antimicrobial susceptibility testing

**Fig. 2:**
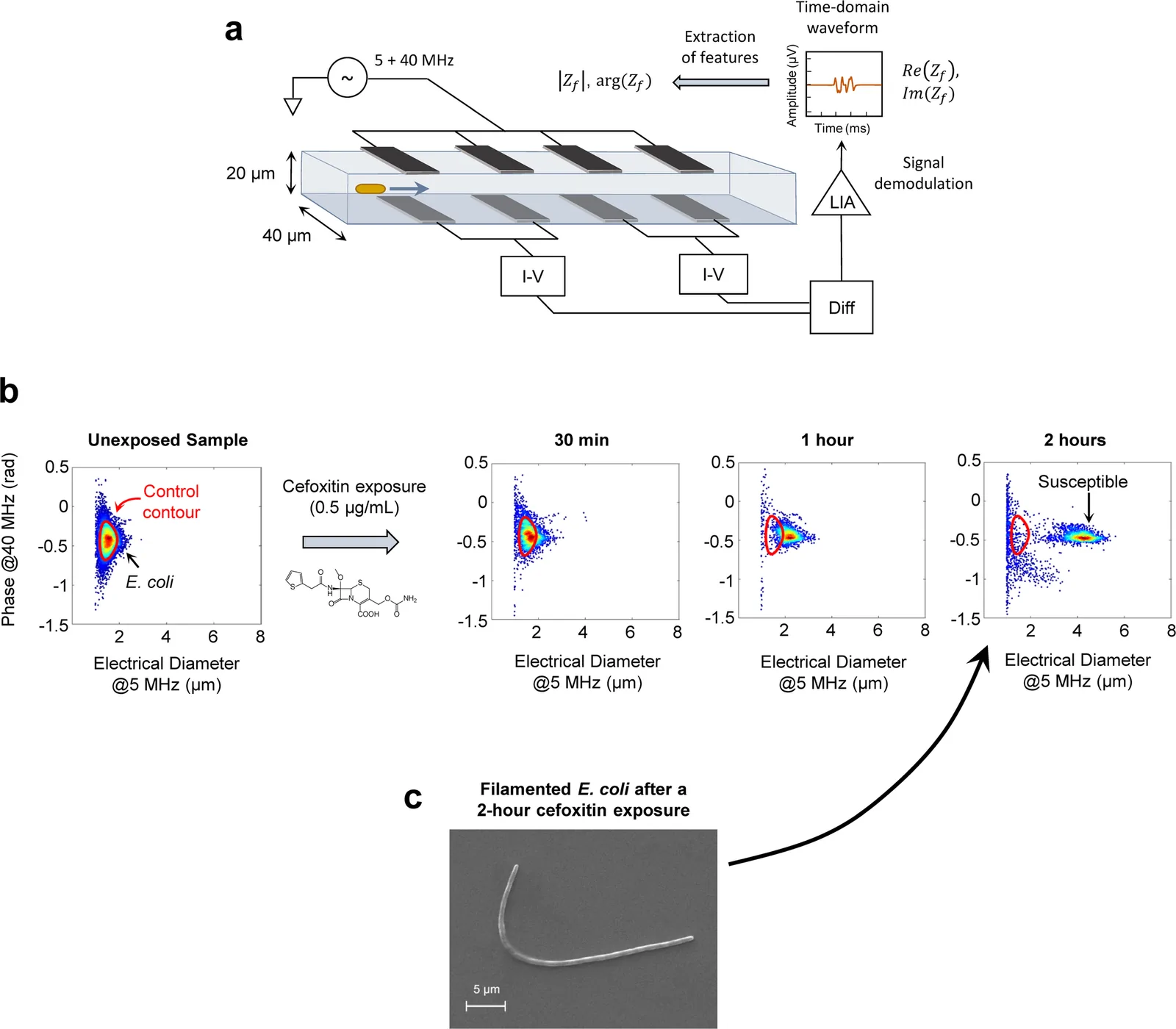
Overview of the impedance cytometry system showing the microfluidic chip and custom electronics used to measure the electrical properties of bacteria. (**a**) Organisms flow through a microchannel containing an array of microelectrodes driven with an AC voltage at 5 MHz and 40 MHz. The electrical current is measured to give the impedance of individual cells which changes after exposure to antibiotics. (**b**) Example impedance scatter plots showing the effect of cefoxitin at 0.5 µg/mL on *E. coli* after 0.5 - 2 h of exposure. (**c**) The SEM image shows a filamented *E. coli* after a 2 h exposure to cefoxitin

Microfluidic impedance cytometry is a high-throughput, label-free technique that measures the electrical properties of thousands of individual cells (Gawad et al. 2004, Sun and Morgan 2010, Honrado et al. 2021). In an impedance cytometer, cells flow along a microfluidic channel within which are arrays of microelectrodes as shown in Fig. 2(a). An AC signal is applied to one set of electrodes, and the current is measured from the opposite set of electrodes using bespoke electronics. As the cells flow along the channel the electrical current is perturbed, and the small change is measured and processed to determine its electrical impedance. The microfluidic channel is approximately 20 μm × 40 μm and the electrodes are driven with two signals at 5 MHz and 40 MHz. At low AC frequencies (5 MHz), the impedance signal measures the cell volume, at higher frequencies the signal is influenced by the cell membrane and cell wall, and to a lesser extent the properties of the cytoplasm. If for example a bacterial cell membrane becomes electrically leaky following exposure to antibiotics, then the electrical signal will change leading to a measurable difference in the cell impedance signature (Spencer et al. 2020, Morgan et al. 2024). Data is plotted as a scatter plot of individual signals from several thousand bacterial cells, as shown in the figure, where electrical phase @40 MHz is plotted against the magnitude of the electrical impedance signal @5 MHz. Figure 2(b) shows an example of an impedance scatter plot for *E. coli* exposed to the antibiotic cefoxitin (a β-lactam antibiotic). After 2 h the electrical properties of the bacteria have changed (increase in electrical volume) as shown in the scatter plots, indicating that these organisms are susceptible to cefoxitin at this concentration. This data is consistent with the morphological changes that occur in *E. coli* following exposure to this antibiotic where cells form long filaments (Zimmerman and Stapley 1976) as shown by the SEM image in Fig. 2(c). This technique can be used to perform a rapid impedance-based AST as in the iFAST method (Hornsey et al. 2026).

## Methodology

### Blood cultures

*Escherichia coli* 25922 and *Staphylococcus aureus* 29213 were obtained from American Type Culture Collection (ATCC). For the experiments, stock samples were removed from − 80 °C storage and cultured overnight on Trypticase Soy Agar (TSA) plates. Single colonies were picked and suspended into Mueller-Hinton Broth (MHB) to prepare an inoculum of the order 10^3^ Colony-Forming-Units per millilitre (CFU/mL). One millilitre of the inoculum was mixed with 8–10 mL of healthy human blood (provided by the Southampton Research Biorepository) and incubated overnight in BD BACTEC aerobic blood culture bottles. The blood-to-broth media ratio inside each bottle was approximately 1:3 v/v. Positive control samples were prepared using the same bottles containing only the broth and the same inoculum of bacteria held in MHB. Negative control samples were also prepared using blood and broth (1:3 v/v) but without bacteria. The BACTEC aerobic bottles contain resin beads to neutralise antibiotics that may be found in patient blood, but no antibiotics were used in this work.

### Lysis buffers and extraction protocols

Five different protocols were used as detailed below and summarised in Table 1. Protocol **1** is the commercially available MBT Sepsityper^®^ kit (Bruker Daltonics), which has a proprietary lysis buffer containing an ionic detergent such as Sodium Dodecyl Sulphate (SDS) (Weller and Maier 2011, Tanner et al. 2017). This kit is widely used to isolate bacteria for MALDI-TOF ID (Morgenthaler and Kostrzewa 2015, Ponderand et al. 2020, Schubert et al. 2011, Tanner et al. 2017, Choi et al. 2025, Lin et al. 2022). Protocol **2** uses the non-ionic detergent saponin (Lee et al. 2018) to permeabilise RBC membranes (Baumann et al. 2000). Protocol **3** follows Boardman et al. 2015 with ammonium chloride (NH_4_Cl) and potassium bicarbonate (KHCO_3_) to lyse RBCs. The NH_4_Cl buffered in KHCO_3_ accelerates ammonium transport into the RBC raising the internal osmotic pressure leading to lysis (Sudnitsyna et al. 2016). Protocol **4** uses a well-established RBC lysis method where deionised water (DI H_2_O) lyses cells by hypoosmotic shock, for example (Boyd et al. 2015). Protocol **5** is based on van Assche et al. 2020 and uses saponin together with Triton X-100, which creates membrane pores leading to cell lysis (Srokowski and Woodhouse 2017). Reducing agents, including sodium thioglycolate and L-cysteine, are added to the buffer to prevent the accumulation of Reactive Oxygen Species (ROS) after RBC lysis (Dutra et al. 2014), which can accelerate bacterial cell death (Hong et al. 2019).

For analysis of extracted bacteria, samples were resuspended in MHB and incubated at room temperature for 20 min prior to dilution in normal saline for measurement with impedance cytometry. An overview of the entire experimental method is shown in Supplementary Fig. 1, and detailed workflows for each protocol are in Supplementary Fig. 2.

**Table 1:** Summary of the five different blood lysis and centrifugation protocols used in this work

Protocol name (adapted from)	Lytic mechanism	Reagents	Centrifugation	Hands-on time	Estimated total duration
1. Sepsityper^®^	Ionic detergent	0.2 mL, 1:5 v/v lysis buffer (proprietary) to PBC 1 mL, 1:1 v/v washing buffer to initial PBC volume	2 × 16,000×g (3 min each)	10 min	16 min
2. Saponin (Lee et al. 2018)	Non-ionic detergent	Saponin (1% w/v), 1:1 v/v buffer to PBC DI H_2_O (1:1 v/v with PBC)	2 × 5,000×g (3 min each)	10 min	16 min
3. Boardman (Boardman et al. 2015)	Ammonium mediated osmotic stress	NH_4_Cl/KHCO_3_ (50/10 mM), 2:1 v/v buffer to PBC CHAPS (1.75 mM), 1:1 v/v buffer to initial PBC volume	3 × 3,200×g (20 min each)	15 min	75 min
4. DI H_2_O (Boyd et al. 2015)	Hypoosmotic	1:1 v/v DI H_2_O to PBC	150 g for 1 min 16,000×g for 2 min	5 min	8 min
5. Van Assche (van Assche et al. 2020)	Non-ionic detergent	1 mL of lysis buffer: 0.7 mg/mL L-cysteine + 0.4 mg/mL sodium thioglycolate + 0.25% w/v saponin + 0.03% v/v Triton X-100 1:1 v/v lysis buffer to PBC (added twice consecutively)	2 × 2,200×g (10 min each)	15 min	35 min

### Cell viability

Short-term viability of bacteria extracted with protocols **1** and **2** was determined over a 3-hour period with live/dead fluorescence staining and flow cytometry. Extracted samples following the two protocols were also measured separately using impedance cytometry. The viability and culturability of the bacteria was evaluated by standard overnight culture on TSA media, the colonies formed after incubation were counted to determine the absolute counts after extraction. A modified Miles and Misra method (M&M) was used for this (see Supplementary Fig. 3(b)). The extraction efficiency (%) of viable bacteria was also estimated immediately after extraction with impedance cytometry by comparison with the M&M counts.

### Impedance flow cytometry analysis and gating

All culture suspensions, including negative and positive controls were diluted 1,000 to 10,000-fold into physiological saline (~ 1.45 S/m) that also contained a suspension of 2 μm diameter calibration beads at ~$\:1\times\:{10}^{6}$ /mL. Approximately 250 µL of each sample was drawn into a syringe and pumped through the impedance chip at a flow rate of 30 µL/min. The impedance signals were sampled at 230k samples/sec and saved into structured data files. Signals were processed and analysed using MATLAB (The Mathworks Inc., R2025a). Scatter plots were generated from the raw impedance data and in each plot a gate was drawn manually to create a bounding box. A non-parametric representation of a probability density function (Kernel Distribution) was applied to the clusters of bacteria identified within this box. The function was used to automatically generate a contour to further refine the gate. The number of bacteria within the gate was counted and converted to an absolute concentration in CFU/mL using the sample dilution factor, flow rate and run time through the impedance cytometer. GraphPad Software (2026) was used to calculate unpaired t-tests comparing the counts from impedance cytometry and M&M.

## Results

Bacteria must be counted to prepare an inoculum for a phenotypic AST assay, such as broth microdilution. EUCAST recommends an inoculum of $\:5\times\:{10}^{5}$ CFU/mL, and this concentration is also used in the iFAST method (Hornsey et al. 2026). In clinical laboratories, a 0.5 McFarland standard is routinely used to create a stock suspension requiring further dilution to reach the EUCAST inoculum concentration. However, when blood cells and debris are present, the McFarland method becomes unreliable because these particles increase the turbidity of the suspension and skew the measurement. With impedance cytometry, bacterial cells or aggregates may be discriminated from RBCs and cellular debris remaining after blood lysis. Each bacterial event can then be treated as one counting unit (or CFU), allowing accurate inoculum preparation for an AST.

### Analysis of blood culture samples

The BD BACTEC FX incubator measures the increase in CO_2_ associated with the active metabolism of bacteria. A fluorescence indicator is inside each bottle, and the incubator measures the fluorescence intensity periodically. Supplementary Fig. 3(a) shows plots of fluorescence intensity vs. time for all cultures. The blood bottle incubator flags positive when there is a significant increase in fluorescence. As shown in the figure, all blood bottles spiked with bacteria flagged positive within 7–9 h from the time of incubation. Sample processing commenced 6–8 h later, giving a total incubation time of 15 h, similar to routine diagnostic workflows (Savinelli et al. 2004). A blood-to-culture media ratio of approximately 1:3 v/v also reflects the typical conditions used in a clinical diagnostic laboratory.

**Fig. 3:**
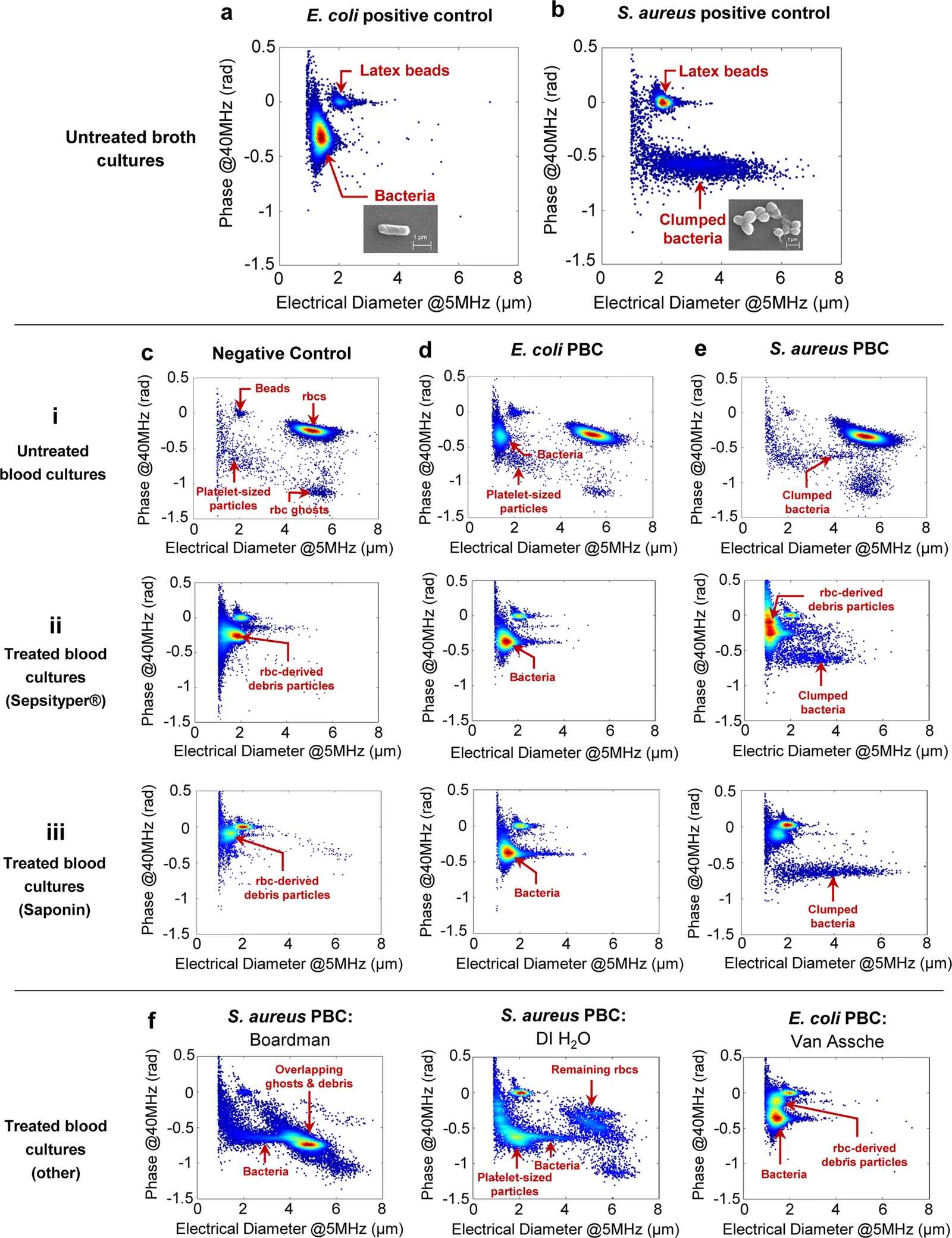
Impedance scatter plots for positive/negative controls and PBCs before and after treatment with the different lysis protocols. (**a**) & (**b**) Scatter plots of positive controls (broth cultures) for *E. coli* and *S. aureus*. (**c**)(i), (**d**)(i) & (**e**)(i) Scatter plots of untreated blood cultures including the negative control, *E. coli* PBC and *S. aureus* PBC, respectively. Scatter plots (**c**)(ii)-(iii), (**d**)(ii)-(iii) & (**e**)(ii)-(iii) are from the same blood cultures after treatment with the Sepsityper^®^ and Saponin protocols. (**f**) Scatter plots of positive blood bottle samples extracted with the Boardman (left), DI H_2_O (middle), and Van Assche (right) protocols

Figures 3(a) and 3(b) show example impedance flow cytometry scatter plots for the positive control samples, i.e. bacteria cultured in blood bottles containing broth but no blood. Each plot shows a population of 2 μm reference beads used to normalise the impedance data, with electrical phase set to zero radians. In each scatter plot there is a clear single population of bacteria. For the same inoculum and growth period, the *E. coli* population outnumbers *S. aureus* by an order of magnitude. The mean electrical diameter of *E. coli* is 1.38 ± 0.15 μm; *S. aureus* is larger at 3.15 ± 0.84 μm, which may reflect the fact that *S. aureus* aggregates and forms small clusters; compare SEM images in Fig. 3(a) and 3(b). The plots also show a different electrical phase for the two species, with *S. aureus* at a lower phase (− 0.58 ± 0.06 rad) than *E. coli* (− 0.31 ± 0.12 rad). The difference in the high frequency electrical phase reflects differences in the cell wall/membrane structures of the two organism types. This demonstrates that impedance cytometry can discriminate between them and could be used as an electrical analogue of conventional Gram-staining.

Figure 3(c)(i) shows an example impedance scatter plot for negative control blood culture (no bacteria) incubated at 37 °C for 15 h, with the sample directly diluted in saline before impedance cytometry measurement. Several populations are visible in the scatter plot: 2 μm calibration beads, along with RBCs and RBC ghosts (lower phase) (Osaid et al. 2026, Spencer and Morgan 2020) both with a diameter of 5.5 μm consistent with previous electrical measurements of RBC and ghost with a volume of approximately 90 fL (Spencer and Morgan 2020). A significant amount of debris particulate is also present in the size range between 1 μm (the limit of detection) and 3 μm, possibly platelets and RBC fragments.

Figure 3(d)(i) and 3(e)(i) show similar plots for positive blood bottles spiked with *E. coli* and *S. aureus*. This data is from aliquots from each PBC that have been diluted and resuspended in saline without any further processing (no lysis or centrifugation). The bacteria are clearly visible in the *E. coli* PBC, along with large numbers of RBCs and fewer ghosts. The *S. aureus* population is less clear with fewer organisms because they do not grow as quickly as *E. coli* and the RBCs outnumber them. Due to coincidence, the impedance signal from the larger RBCs in untreated samples mask those of bacteria resulting in fewer bacteria being counted.

Figures 3(c)(ii)-(e)(ii) show data for the same samples after extraction using the Sepsityper^®^ protocol. The negative control (no bacteria) contains a large population of particles in the same size range as *E. coli* (1–2 μm), possibly small ghost cells and large (multi-lamellar) vesicles that form during detergent lysis. For the Sepsityper^®^ protocol, the *E. coli* appear as a distinct population (Fig. 3(d)(ii)) because the very high number density of bacteria masks the debris/particles. However, the *S. aureus* population is more difficult to identify because there are fewer bacteria and in this case the debris outnumber the bacteria. Although the *S. aureus* population is visible in this particular example, this varies considerably from sample to sample (see later).

Figure 3(c)(iii)-(e)(iii) show data for the Saponin-extracted samples (protocol **2**). The negative control shows a similar population to the Sepsityper^®^-treated sample, but there are fewer debris particles. Likewise, the extracted *E. coli* PBC is also distinct and is indistinguishable from the Sepsityper^®^-treated PBC sample. The *S. aureus* bacteria are visible as a distinct population because the debris is smaller and does not overlap with the bacteria.

Fig. 3(f) shows representative data for samples extracted using the three remaining protocols listed in Table 1. The Boardman protocol produces a large amount of cellular debris that overlaps with the *S. aureus* population. The dense population in the plot consists of RBC ghosts formed after osmotic lysis. For this reason, it is impossible to count the entire population of bacteria and produce an accurate CFU concentration as required for a phenotypic AST inoculum. Similarly, deionised H_2_O lysis (protocol **5**) produces a smaller population of non-bacterial particles around 2 μm in diameter that also overlap with the *S. aureus* population (possibly platelets). Some intact RBCs also remain, presumably because insufficient cell lysis occurred at the ~ 1:1 v/v supernatant-to-water dilution ratio, which is close to the threshold for osmotic lysis (~ 165 mOsm).

The rightmost scatter plot in Fig. 3(f) shows data for *E. coli* extracted with the Van Assche protocol where there is an overlap of debris with the *bacteria* population. The addition of Triton X-100 (0.03% v/v) alongside saponin (0.25% w/v), combined with lower centrifugation speeds (2200 × g), appears to retain a greater number of particles around 1.5 μm in electrical diameter compared with saponin alone at 1% w/v and higher centrifugation speeds (5000 × g). However, *E. coli* reaches very high concentrations in blood culture bottles after 15 h of incubation so the organisms can be discriminated from the debris. These results suggest that blood lysis and centrifugation may not even be necessary for *E. coli* and perhaps other Gram-negative bacteria that grow quickly in PBCs.

### Growth kinetics

**Fig. 4:**
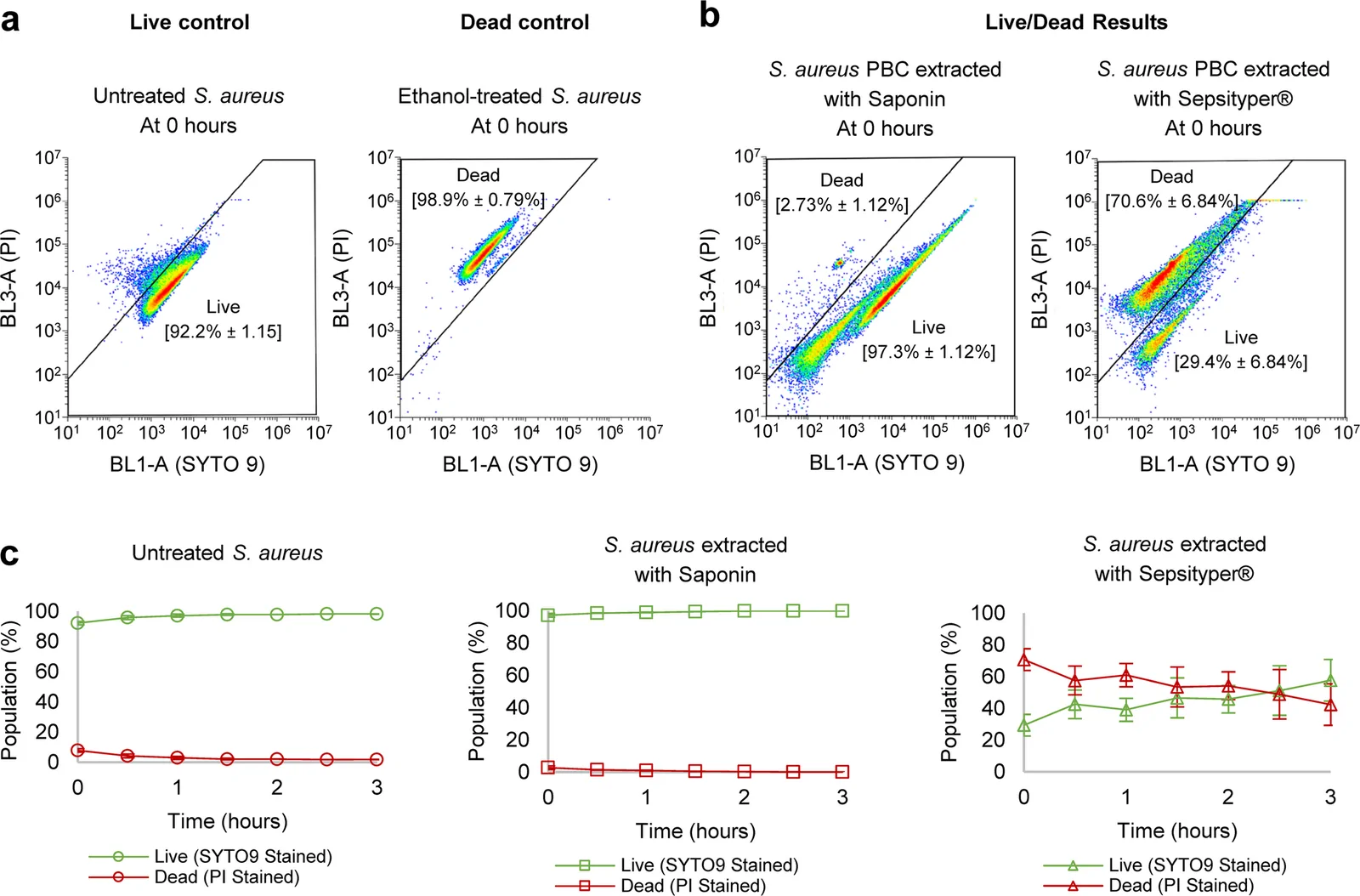
Optical flow cytometry data using Live/Dead staining assay. (**a**) Scatter plot of PI vs. SYTO-9 fluorescence for *S. aureus* with the boundary used to define the “Live/Dead” gates. (**b**) Scatter plots for the Saponin and Sepsityper^®^-treated cells immediately after extraction (t = 0 h). (**c**) % of “Live vs. Dead” cells as a function of time for cells resuspended in MHB at 37 °C and measured every 30 min (*n* = 3)

Gram-negative bacteria can be extracted from blood using detergent lysis with minimum impact on viability (Coskun et al. 2025). However, viable Gram-positive bacteria such as *Streptococcus pneumoniae* and *Staphylococcus aureus* are more difficult to isolate, and extraction protocols have a significant impact on viability (Choi et al. 2025, Coskun et al. 2025). In this work, the viability of *S. aureus* was examined after detergent extraction by staining cells with fluorescent viability dyes (BacLight™) and analysis with optical flow cytometry (Thermo Fisher Attune NxT). Two dyes were used: SYTO 9, a green nucleic acid stain that labels both viable and membrane-compromised cells, and propidium iodide (PI), a red dye that penetrates only bacteria with damaged membranes. These are combined to give a “Live/Dead” assay. For each individual biological experiment, the *S. aureus* grown in broth without blood was used to define the “Live” cell gate, and a second sample of this same culture was treated with 70% ethanol to induce membrane damage and define the “Dead” cell gate (see Fig. 4(a)). Bacteria were stained according to the BacLight™ kit protocol and for each sample measurement approximately 20,000 events were recorded. Fluorescence thresholds in the flow cytometer were kept constant to ensure accurate gating of each population. Untreated live control samples (no lysis or centrifugation) from positive bottles, together with processed positive blood bottle samples using only the Sepsityper® and Saponin lysis protocol (protocols **1 **and **2**) were first labelled with the dye, then suspended in MHB and kept in an incubator at 37 °C then later analysed with optical flow cytometry. Scatter plots of PI vs. SYTO 9 emission were generated using FlowJo.

Figure 4(a) and 4(b) show data from samples immediately prior to incubation (t = 0 h) for the two different detergent extraction protocols. Bacteria were measured every 30 min to determine the proportion of viable (“Live”) and non-viable (“Dead”) cells, with the data summarised in Fig. 4(c) as the percentage of total cell counts as a function of time.

Figures 4(a) and 4(b) show that viable and non-viable cells can be discriminated with the fluorescent dyes. After detergent extraction most of the *S. aureus* cells isolated using Sepsityper^®^ are non-viable (strong PI signal), which is not seen for the Saponin-treated cells. The time-course data shows that Saponin-treated cells are unaffected and recover as quickly as the control (untreated) cells. The data for the Sepsityper^®^-extracted cells shows that they recover only after several hours. The Sepsityper^®^ kit has a proprietary lysis buffer that may contain SDS, as suggested by the patent (Weller and Maier 2011). SDS is an anionic detergent that can permeabilise bacterial membranes (Kennedy et al. 2011). This may explain the increased PI signal observed after treatment with Sepsityper^®^. Whether SDS also causes oxidative stress remains unclear, however Song et al. (2018) shows that an ionic surfactant (bis-quaternary ammonium salt) can increase intracellular ROS in *S. aureus*.

To further assess viability and growth of *S. aureus* after isolation, the electrical properties of the bacteria were measured with impedance cytometry. Triplicate measurements were taken every 30 min over a 3-hour window. Untreated positive blood culture samples were used as controls. The viability of *E. coli* was also measured in this way, although these organisms are much less affected by exposure to detergents.

**Fig. 5:**
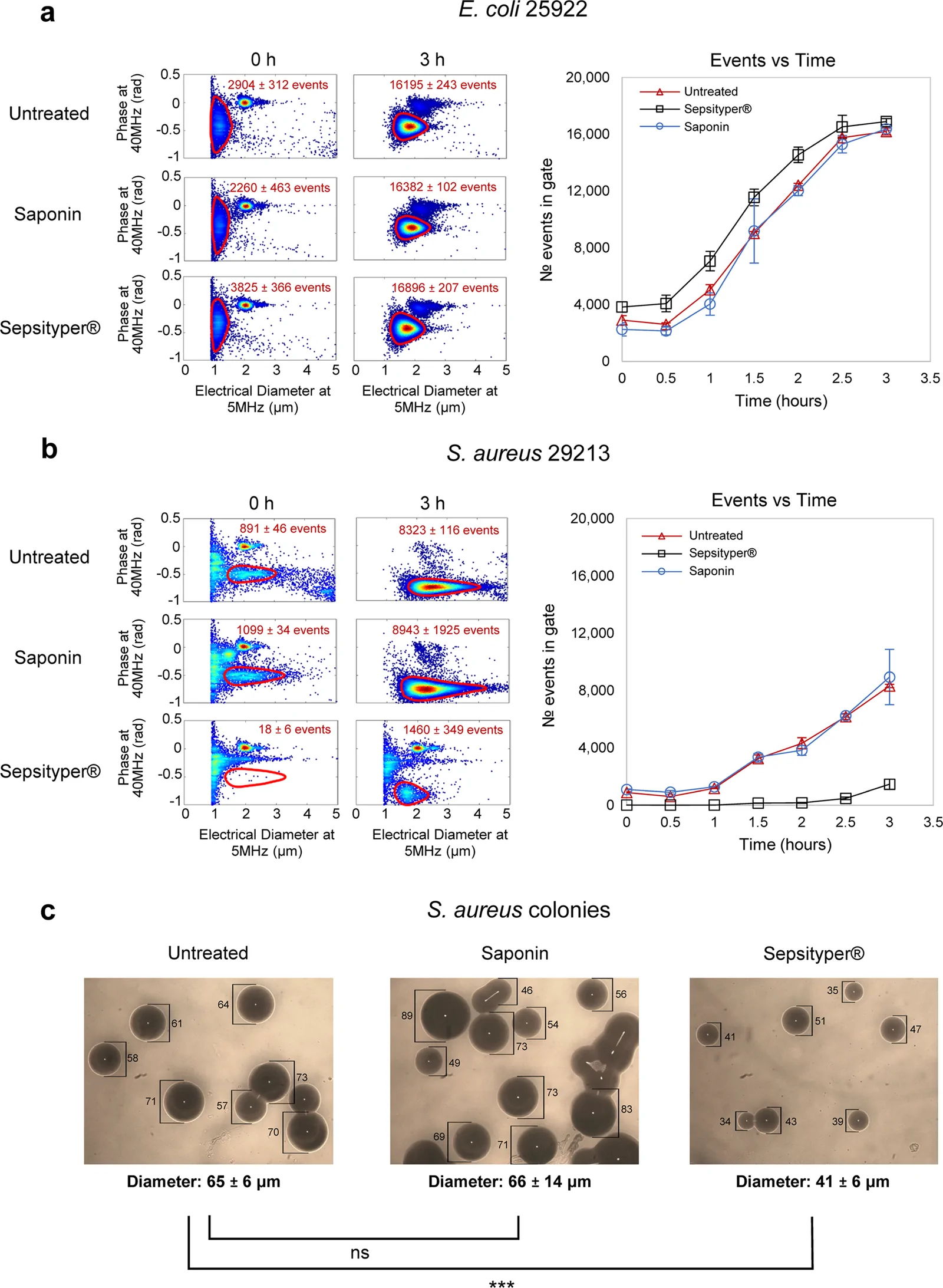
Time-course impedance scatter plots of untreated bacteria, and after extraction from a positive blood bottle with Saponin and Sepsityper^®^. (**a**) *E. coli* and (**b**) for *S. aureus*. The number of cells within the red contour is shown on the plot with mean and standard deviation of three replicates. (**c**) Optical microscope images of colonies before (untreated) and after extraction from positive blood bottles. The images were taken after 18 ± 2 h of growth on agar plates. The numbers in the images correspond to colony diameter (µm); with mean and standard deviation shown. Statistical significance values are reported as: *p* > 0.05 (ns) and *p* < 0.001 (***). Images were acquired using a digital camera mounted on a Nikon Eclipse LV100 microscope with a 5x objective lens

Figure 5(a) and 5(b) show impedance scatter plots for *E. coli* and *S. aureus* after extraction with Sepsityper^®^ and Saponin at two time-points: immediately after extraction (0 h) and at 3h. Bacteria were counted using the gating method outlined in the Methodology section. At 3 h the cell volume for *E. coli* has increased for all three data sets, with the mean diameter changing from 1.15 μm to 1.82 μm. There is also a small shift in electrical phase, from − 0.36 rad to − 0.42 rad. The number of measured *E. coli* events for each sample is also plotted as a function of time showing a short growth delay for 30 min followed by normal growth. The larger number of bacteria in the Sepsityper^®^-treated *E. coli* samples leads to a vertical offset in the time-course at 0 h due to a slightly greater number of events in this case, but the growth rate is similar in the three curves.

For *S. aureus*, Fig. 5(b) shows that there is likewise a short growth delay (1 h in this case) followed by normal growth for both the untreated and Saponin-treated samples, whereas the Sepsityper^®^-treated samples only start to show growth after 2.5 h, in agreement with the fluorescent data in Fig. 4(c). The untreated and Saponin-treated samples also show a similar change in electrical properties after 3 h: the untreated population shows a diameter increase from 2.08 μm to 2.43 μm, together with a shift in phase magnitude (− 0.52 rad to − 0.75 rad), and the Saponin-treated population from 2.01 μm to 2.14 μm, and − 0.53 rad to − 0.78 rad. The progressive shift in phase during growth may reflect differences in the electrical phenotype of the bacterial envelope as the cells transition into active division. At 0 h, Sepsityper^®^-extracted *S. aureus* could not be identified at a dilution of 1:1,000 (sample-to-saline v/v) indicating a low number of viable organisms after extraction. The bacteria begin to appear at 3 h as a population with a small cell volume (1.72 μm diameter).

Note that the curves saturate at higher cell concentrations because of coincidence in the impedance cytometer, rather than a reduction in growth rate. From these curves the doubling times were estimated by a line of best fit in the linear range as 34 ± 1 min (untreated), 40 ± 4 min (Sepsityper^®^) and 31 ± 5 min (Saponin) for *E. coli*. For *S. aureus*, these were 65 ± 5 min (untreated) and 67 ± 8 min (Saponin). It was not possible to extract a doubling time for *S. aureus* after Sepsityper^®^ extraction because the organisms only started to grow after 2.5 h. The doubling times appear slightly longer than those typically reported for organisms grown under optimal laboratory conditions in nutrient-rich broth without blood. However, the differences between the untreated (no detergent exposure) and saponin-treated samples were not statistically significant for either the Gram-negative or Gram-positive organisms (*p* = 0.5 and *p* = 0.8, respectively). Furthermore, the bacteria reach exponential growth within a 2-hour period, showing compatibility of the extraction protocol with the iFAST method. 

Figure 5(c) shows images of *S. aureus* colonies grown on TSA plates for 18 ± 2 h at room temperature. Colony diameters were measured for each of the three samples. No significant difference in diameter was observed between untreated and Saponin-treated colonies (*p* > 0.05), whereas colonies isolated with Sepsityper^®^ were significantly smaller than those from the untreated PBC sample (*p* < 0.001) indicating impaired growth.

The results from optical cytometry, impedance cytometry, and colony images demonstrate that isolation of *S. aureus* following Saponin treatment did not unduly affect growth and may be suitable for isolation of viable cells. This contrasts with isolation of *S. aureus* with Sepsityper^®^ where growth was inhibited for 2 to 3 h. No delay in growth was observed for *E. coli* for either of the two protocols.

### Extraction efficiency

**Fig. 6:**
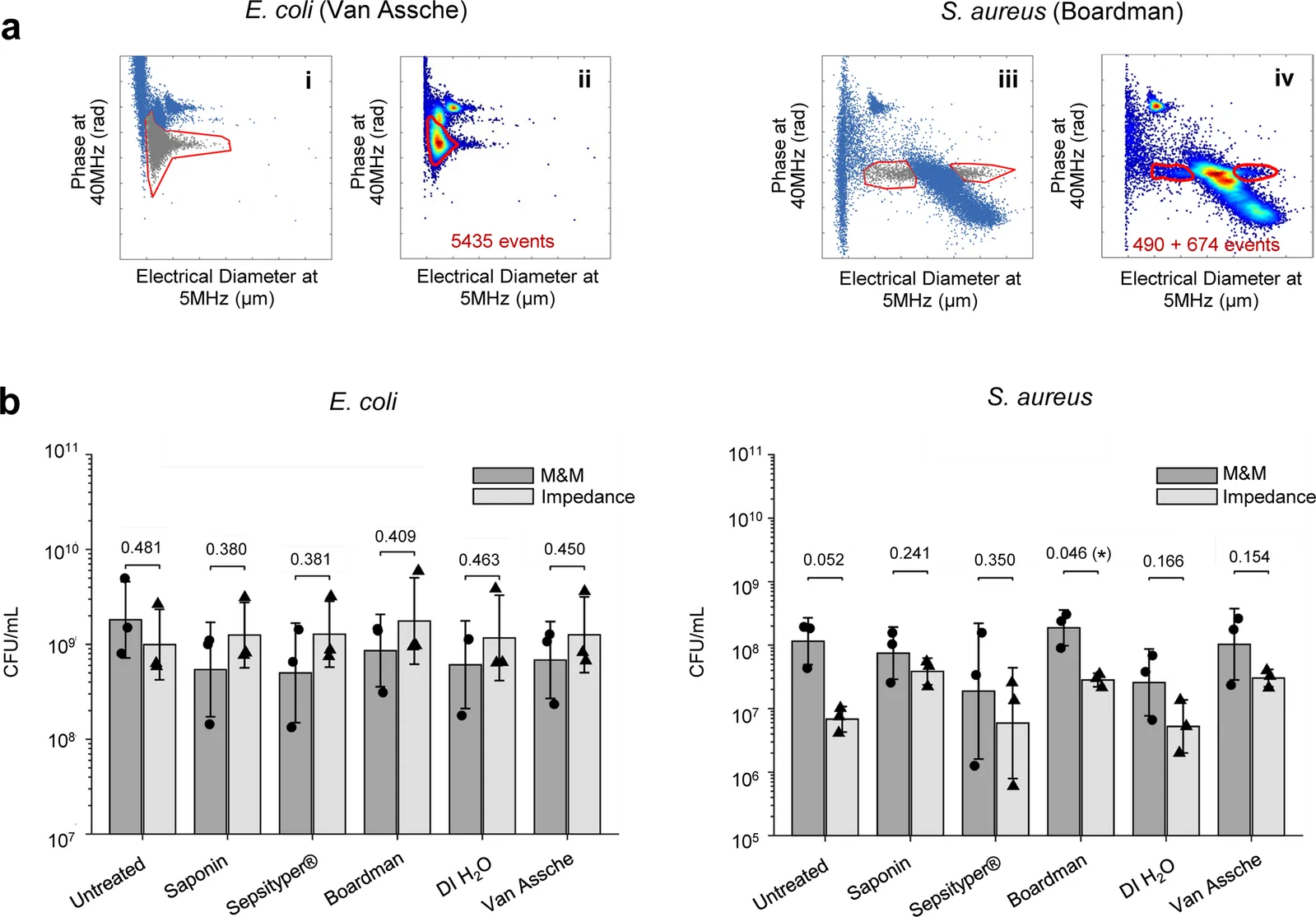
Summary of recovery after isolation from positive blood bottles using the five different extraction protocols. (**a**) A coarse bounding box drawn manually (i) and (iii) that is further refined with an automated contour (ii) and (iv). (**b**) Absolute counts (CFU/mL) for *E. coli* and *S. aureus*, from M&M colony counting and impedance cytometry. Three experiments were carried out for each protocol. Statistical significance is *p* < 0.05 (*) for comparisons between the two methods. P-values are shown on top of each pair

To determine extraction efficiency, bacteria need to be accurately discriminated from the debris remaining after lysis and centrifugation. Example scatter plots for the Van Assche and Boardman extraction methods are shown in Fig. 6(a) (data for other methods are in Supplementary Fig. 4). To distinguish bacteria from debris, a population of bacteria was first defined with a manually drawn bounding box as shown in Fig. 6(a) panel (i) and (iii). A contour was then automatically generated to refine the bounding box (see panels (ii) and (iv)). In the example shown in Fig. 6(a) the Van Assche protocol creates debris similar in size to the bacteria but with a different phase which allows bacteria to be discriminated. For *S. aureus* the Boardman protocol creates much more debris that almost completely overlaps with the bacteria (panels (iii) and (iv)). This means fewer events could be identified as bacteria.

Figure 6(b) compares impedance cytometry counts for all methods immediately after extraction compared with M&M counts after 18 ± 2 h. For *E. coli* there was no significant difference to M&M (*p* > 0.05 for all methods), indicating that most bacteria could be discriminated from debris, and were viable and culturable after extraction. For *S. aureus*, impedance counts were similar to M&M counts for all extraction protocols apart from the Boardman protocol (*p* < 0.05) and untreated samples (*p* = 0.05), due to an overlap with a large amount of debris (Fig. 6(a) panel iv), or coincidence with RBCs during analysis of the untreated samples (refer to the previous Fig. 3(e)(i)).

*E. coli* counts were in the range of $\:{5\times\:10}^{8}$ to $\:2.5\times\:{10}^{9}$ CFU/mL; and one order of magnitude lower for *S. aureus* reflecting the greater number of *E. coli* in the PBC. It appears that fewer *E. coli* events were measured by impedance for the untreated PBCs (not statistically significant), but this is because of coincidence with the RBCs and ghosts. Detergent lysis and centrifugation removed these particles, reducing coincidence (refer to Fig. 3(e)(ii)). This was also observed for untreated *S. aureus* PBCs, but for this organism the Sepsityper^®^ and DI H_2_O protocols were less efficient than for *E. coli*. For the Sepsityper^®^ protocol, the debris is well separated from the bacteria; the lower counts are probably a result of growth inhibition due to detergent exposure. Low impedance counts following DI H_2_O extraction may be due to sample loss during the first protocol step (see Table 1), possibly because *S. aureus* sedimented with the RBCs as they are known to adhere to them (Shin et al. 2005). This was not observed with *E. coli*. For Boardman and Van Assche cell counts determined with M&M slightly exceeded the untreated *S. aureus* counts, possibly due to clumps of bacteria disaggregating during the blood lysis and centrifugation procedures, allowing more colonies to grow.

Extraction Efficiency (* EE*) was calculated from M&M colony counts and impedance cytometer counts for each biological replicate (*n* = 3; Table 2). The M&M ratio represents the proportion of viable and culturable bacteria recovered after extraction relative to the corresponding untreated PBC:

$$\:{EE}_{M\&M\left(\%\right)}=\:\frac{{N}_{M\&M}\left(Extracted\right)}{{N}_{M\&M}\:\left(Untreated\right)}\times\:100$$

where $\:{N}_{M\&M}$ is the CFU/mL determined by M&M colony counting.

The number of events counted with impedance cytometry ($\:{N}_{Z}$) after extraction compared to the viable CFU/mL of the respective untreated PBC was also determined as

$$\:{EE}_{Z\left(\%\right)}=\:\frac{{N}_{Z}\left(Extracted\right)}{{N}_{M\&M}\:\left(Untreated\right)}\times\:100$$

For *E. coli*, $\:{EE}_{Z}$ was consistently higher than $\:{EE}_{M\&M}$ (67–101% vs. 37–54%, respectively), although none of the differences were statistically significant. The Boardman protocol produced the highest EE values in both methods for *E. coli*.

For *S. aureus*, $\:{EE}_{Z}$ was consistently lower than $\:{EE}_{M\&M}$. The greatest difference was observed after Boardman extraction (28% and 166%, respectively; *p* = 0.005). Values exceeding 100% are probably due to dissociation of aggregates during extraction. Debris within the impedance gate may have also contributed to the increase in $\:{EE}_{Z}$. Saponin produced the highest $\:{EE}_{Z}$ (35%) for *S. aureus*, whereas DI H_2_O produced the lowest values with both measurement techniques (4.9% and 24%). The efficiencies in Table 2 show that all extraction protocols perform well with *E. coli*. However, with *S. aureus*, the Saponin and Van Assche extraction provided the best balance of viable recovery, impedance counts and variability between samples.

**Table 2:** Comparison of$\:{EE}_{M\&M\left(\%\right)}$with$\:{EE}_{Z\left(\%\right)}$for each organism and protocol.$\:\stackrel{-}{x}$is the mean of three biological repeats and$\:\sigma\:$the standard deviation. Statistical significance is reported as: *p* < 0.01 (**).

	*E. coli* (%)					*S. aureus* (%)				
Sample (*n* = 3)	$\stackrel{-}{x}$(EE_M&M_)	*σ*(EE_M&M_)	$\stackrel{-}{x}$(EE_Z_)	*σ*(EE_Z_)	*p*-value	$\stackrel{-}{x}$(EE_M&M_)	*σ*(EE_M&M_)	$\stackrel{-}{x}$(EE_Z_)	*σ*(EE_Z_)	*p*-value
Saponin	37	± 26	72	± 27	0.215	65	± 11	35	± 14	0.054
Sepsityper^®^	42	± 39	74	± 31	0.366	55	± 38	15	± 15	0.237
Boardman	54	± 28	101	± 33	0.158	166	± 32	28	± 19	0.005 (**)
DI H_2_O	40	± 25	67	± 21	0.279	24	± 10	4.9	± 2.4	0.050
Van Assche	45	± 28	74	± 29	0.340	96	± 33	29	± 17	0.061

## Discussion & conclusion

Rapid sample preparation methods involving detergent lysis and centrifugation are widely used to isolate bacteria from PBCs for identification using MALDI-TOF ID. For rapid phenotypic AST the viability and culturability of bacteria is important. Gram-positive pathogens, such as *S. aureus,* account for more than half of bloodstream infections (Pfaller et al. 1999, Mendes et al. 2018) but are more susceptible to damage after exposure to the detergents commonly used for RBC lysis. In this work we investigated the viability and culturability of two representative organisms, *E. coli* and *S. aureus *after processing from positive blood bottles containing healthy human blood using five different blood lysis protocols.

Fluorescent viability dyes showed that the widely used Sepsityper^®^ lysis method causes damage to *S. aureus* inhibiting growth by approximately 2 h, but with limited impact on the Gram-negative *E. coli.* Debris generated during extraction may interfere with bacterial counts and bias the inoculum in an automated AST. Samples were therefore analysed by impedance cytometry and standard agar plate colony counting (M&M). Absolute counts determined with impedance cytometry were in overall agreement with the reference M&M method for *E. coli*. For *S. aureus*, the outcome was different owing to the slower growth rate of *S. aureus* and the presence of fewer organisms in the PBC bottles at positivity. The number of viable bacteria extracted from PBCs for *E. coli* was in the range 37% to 54%; lower for *S. aureus* (5% to 35%). Saponin did not inhibit the growth of *S. aureus* at a concentration of 1% (w/v) and produced the cleanest samples with the highest extraction efficiency.

Future work will focus on streamlining the sample preparation method into an automated microfluidic sample processing system. For example, use of continuous flow on-chip lysis (Holmes et al. 2009) with automated washing could be used for the first steps, followed by size fractionation using techniques such as inertial, viscoelastic or electrokinetic methods. Further work will also explore the utility of the method with clinical samples, and a broader range of Gram-positive pathogens. In some cases, clinical blood may contain more than one organism, and polymicrobial samples require pathogen separation or species-specific analysis before phenotypic AST, as in (Kandavalli et al. 2022).

## Supplementary Information

Below is the link to the electronic supplementary material.

Supplementary Material 1

## Data Availability

The source data for the figures can be found at https://doi.org/10.5258/SOTON/D3944.

## Declarations

### Source of Biological Material

The whole blood used in this study was provided by healthy volunteers from the Southampton Research Biorepository (SRB) at the University Hospital Southampton (UHS).
