# Supplementary material for: Isolating viable bacteria from positive blood cultures: impedance cytometry analysis
Source: Biomed Microdevices. 2026 Oct 1;28(4):71. doi: 10.1007/s10544-026-00847-5 (PMC13630967; doi:10.1007/s10544-026-00847-5)
Supplement: Supplementary file 1 [file 10544_2026_847_MOESM1_ESM.docx]

**Supplementary Information**

### **Experimental method**

**Supplementary Figure 1.** The experimental workflow followed in this work.

### **Extraction protocols**

**Supplementary Figure 2.** The extraction protocols used in this work.

### **The modified Miles and Misra method**

The Miles and Misra (M&M) method is widely used to count viable bacteria. It involves the series dilution of a bacterial suspension and pipetting of droplets from each dilution onto solid agar media. The bacteria are incubated overnight to promote the growth of colonies within each droplet. A moderately dense droplet of 10-50 colonies is picked for counting (by eye), and the cell density (CFU/mL) of the initial suspension can be calculated.

In this work, the M&M method was modified by growing colonies for 18 ± 2 hours at room temperature rather than by incubation at 37°C to prevent excessive growth and enable enumeration of moderately sized colonies using a 5x objective lens. A Nikon Eclipse LV100 microscope with an electronic camera was used to take digital images of colonies. A virtual grid of 1 mm^2^ was applied to the images and the number of colonies within the grid area ($A_{g}$) was counted. The area of the circular droplet ($A_{d}$) was measured using a calliper, and this was used to scale the number of colonies in proportion to the droplet area. The virtual grid was applied three times to arbitrary locations inside each droplet, and the average of the three was taken to obtain an unbiased count ($N_{col}$).

The following fraction was used to obtain the viable count:

$${CFU}/{mL}= \frac{N_{col}\cdot V_{s}\cdot A_{d}}{{D_{f}\cdot V}_{d}\cdot A_{g}}$$

where $A_{d}$ is the droplet area, typically ~28mm^2^ for 3 μL, $A_{g}$ is the grid area (1 mm^2^), $D_{f}$ the droplet dilution factor (e.g. ${10}^{-4}$ from stock suspension), $N_{col}$ is the number of colonies counted inside the grid (e.g. 15 colonies), $V_{d}$ is the droplet volume (3 μL), and $V_{s}$ is the volume of the stock suspension (1 mL).

This method enables counting of denser droplets with hundreds to a thousand colonies that would otherwise be impossible to perform by eye, thus improving the counting accuracy. SI Figure 3b shows an example of agar plates and corresponding image of magnified colonies.

**Supplementary Figure 3.** **(a)** Fifteen-hour growth curves of Fluorescence Intensity (y-axis) vs Time (x-axis) for *E. coli* and *S. aureus* cultured in aerobic blood bottles. The Time-To-Detection (TTD) is the moment the bacteria were detected (in hours), and this is highlighted in the BD BACTED FX data for each curve. The mean (scatter points) and standard deviation (shaded area) of three experiments is shown. Blood bottles without bacteria were incubated alongside the others as a control (Neg Ctrl) and did not flag positive. **(b)** Example of the Miles and Misra (M&M) method used in this study. Left images are of Trypticase Soy Agar (TSA) plates with *E. coli* and *S. aureus* depicting growth in the less diluted droplets. The droplets with fewer colonies were imaged (right) for counting.

SI Figure 4 is complementary to manuscript Figure 6(a) reporting data for other extraction methods.

**Supplementary Figure 4.** Gating of bacterial populations after extraction with the remaining lysis protocols. **(a)** *E. coli* and **(b)** *S. aureus*. For each protocol, the bacterial population was manually defined with a bounding box in panels **(i)**, **(iii)**, **(v)**, **(vii)** and **(ix)**. Contours are shown in panels **(ii)**, **(iv)**, **(vi)**, **(viii)** and **(x)**.

Absolute counts determined from impedance scatter plots were calculated using the following equation:

$${CFU}/{mL}= \frac{N_{gate}\cdot DF}{Q_{cyt}\cdot t}$$

Where $N_{gate}$ is the number of events inside the generated contour, $DF$ is the dilution factor of sample-to-saline (v/v), $Q_{cyt}$ is the impedance cytometer flow rate in mL/min, and $t$ is the duration of the measurement (e.g., 0.5 min).
